# Effect of breed and pasture type on methane emissions from weaned lambs offered fresh forage

**DOI:** 10.1017/S0021859615000544

**Published:** 2015-08

**Authors:** M. D. FRASER, H. R. FLEMING, V. J. THEOBALD, J. M. MOORBY

**Affiliations:** Institute of Biological, Environmental and Rural Sciences, Aberystwyth University, Gogerddan, Aberystwyth, SY23 3EB, UK

## Abstract

To investigate the extent to which enteric methane (CH_4_) emissions from growing lambs are explained by simple body weight and diet characteristics, a 2 × 2 Latin square changeover design experiment was carried out using two sheep breeds and two fresh pasture types. Weaned lambs of two contrasting breed types were used: Welsh Mountain (WM, a small, hardy hill breed) and Welsh Mule × Texel (TexX, prime lamb) (*n* = 8 per breed). The lambs were zero-grazed on material cut from recently reseeded perennial ryegrass and extensively managed permanent pasture. In each experimental period, individual *ad libitum* dry matter intake (DMI) was determined indoors following an adaptation period of 2 weeks, and CH_4_ emissions were measured individually in open-circuit respiration chambers over a period of 3 days. Although total daily CH_4_ emissions were lower for the WM lambs than for the TexX lambs (13·3 *v.* 15·7 g/day, respectively) when offered fresh forage, the yield of CH_4_ per unit DMI was similar for the two breed types (16·4 *v.* 17·7 g CH_4_/kg DMI). Total output of CH_4_ per day was higher when lambs were offered ryegrass compared with permanent pasture (16·1 *v.* 12·9 g/day, respectively), which was probably driven by differences in DMI (986 *v.* 732 g/day). Methane emissions per unit DMI (16·4 *v.* 17·7 g CH_4_/kg DMI) and proportion of gross energy intake excreted as CH_4_ (0·052 *v.* 0·056 MJ/MJ) were both higher on the permanent pasture. No forage × breed type interactions were identified. The results indicate that forage type had a greater impact than breed type on CH_4_ emissions from growing weaned lambs. It can be concluded that when calculating CH_4_ emissions for inventory purposes, it is more important to know what forages growing lambs are consuming than to know what breeds they are.

## INTRODUCTION

The livestock sector faces major challenges in reducing its contribution to global environmental problems (Steinfeld *et al.* 2006). At the same time livestock numbers are increasing across the globe in response to rising demand for meat and dairy products, with sheep numbers forecast to increase by 60% to 2050 (Foresight 2010). According to the UK's 2012 greenhouse gas (GHG) inventory returns, agriculture was the source of about 0·44 of total UK emissions of methane (CH_4_), and of this *c*. 0·70 came from livestock enteric sources (mostly ruminants) (DECC 2014). Sheep meat makes up 0·10 of the total meat produced and consumed in the UK, but according to available data sheep account for over 0.16 of the GHG emissions from UK livestock species (DECC 2014). Improving the precision of emission data reporting is essential in order to identify key factors influencing the emission rates from different livestock systems and subsequently assessing the effectiveness of mitigation strategies. The current UK National GHG Inventory reports baseline emissions from sheep to the United Nations Framework Convention on Climate Change (UNFCCC) uses the most simplified approach to accounting (Tier 1) and relies on default emission factors (EFs) published by the Intergovernmental Panel on Climate Change (IPCC) (Dong *et al.* 2006). However, many of the observational data that these have been based upon have been collected from breeds or forages unrepresentative of pastures grazed by sheep within the UK and northern Europe (Murray *et al.* 1978; Ulyatt *et al.* 2002, 2005; Pinares-Patiño *et al.* 2003).

In order to provide a robust basis for generating improved EFs for CH_4_ from sheep, baseline data are required for all stages of the production cycle. However, much of the sheep production in the UK is stratified into systems that utilize smaller, hardier breeds in the hills and heavier, more productive breeds and their crossbreds in the lowlands. It is possible that physiological and/or behavioural differences between different breed types may lead to differences in amounts of CH_4_ emitted. As part of a multi-centre research programme developing revised EFs for sheep and cattle the current experiment investigated the extent to which breed × diet relationships for growing lambs were explained solely by simple body weight and digestibility characteristics of the animal and diet, respectively, with no interactions. It quantified, for the first time, CH_4_ emissions from growing weaned lambs fed on pastures representative of those used in UK sheep production systems.

## MATERIALS AND METHODS

### Experimental design

The work described was conducted in accordance with the requirements of the UK Animals (Scientific Procedures) Act 1986 and with the approval of the Aberystwyth University Animal Welfare and Ethical Review Board. Data were collected in a 2 (breed) × 2 (diet) Latin square cross-over design experiment. Measurements were made on lambs of contrasting breed types: (1) Welsh Mountain (WM, small, hardy, hill breed), and (2) Welsh Mule (WM × Bluefaced Leicester) × Texel (TexX, prime lamb) (*n* = 8 per breed). The diets offered were: (1) herbage cut from an intensively managed ryegrass ley, and (2) herbage cut from an extensively managed, long-term permanent pasture

Eight ewe lambs of each breed type, aged *c.* 3 months old at the start of the experiment in June 2013, were selected from their respective flocks on the basis of live weight (LW) and body condition score (BCS) (MLC 2000) and pastured together on a mixed ryegrass/clover sward until the experiment began. All animals were drenched with an anthelmintic prior to the start of each experimental period. Initially half the lambs of each breed type were grazed together on the ryegrass sward, and the other half on the permanent pasture. Following a minimum of 1 week of grazing at pasture the lambs were housed in group pens and zero-grazed on their respective forages. After a further acclimatization period of at least 1 week they were individually penned for 3 days, during which time feed intake was measured. They were then individually housed in one of four calibrated CH_4_ chambers (Gardiner *et al.* 2015) and data collected for 3 consecutive days from each individual animal. Within a given breed type, animals were assigned randomly to an initial forage treatment. Likewise, during each run animals were assigned randomly to individual CH_4_ chambers. Following completion of the first experimental period the animals were swapped to the other pasture type, and the procedure was repeated.

### Forages offered

Two forages were offered to the lambs: (1) ryegrass, a recently sown ley of monoculture perennial ryegrass (*Lolium perenne*), and (2) a long-term ley of a mixture of grass and forb species. During the zero-grazing period, when animals were inside in group pens, individual pens and CH_4_ chambers, forage was cut from the two pastures using a Haldrup plot harvester (J. Haldrup a/s, Løgstør, Denmark). Forage was cut three times per week and stored in a dark cool room at *c*. 4 °C prior to feeding. The lambs were fed on an *ad libitum* basis, with feed quantities designed to ensure a refusal margin of 0·10–0·15 each day, with two equal portions offered at 09·00 and 16·00 h. Fresh water was continually available.

### Animal measurements

The LW and BCS of the lambs was recorded prior to them entering the CH_4_ chambers and again as they were removed. Methane production was determined by comparing the CH_4_ concentrations in the air entering and leaving the chambers at a known rate of airflow. An eight port single channel CH_4_ gas analyser (MGA3000; ADC Gas Analysis Ltd, Hoddesdon, UK) was used to determine CH_4_ concentrations in ambient air and in the exhaust gas leaving each chamber on a rotational basis. Ambient gas was sampled from points between each pair of chambers. The CH_4_ analyser was set to take a reading for each location at the end of a 3-min sampling interval before moving on to the next source. Air flow data was captured using mini-vane anemometers (MiniVane6, Schiltknecht Messtechnik, Switzerland) at each chamber attached to a data logger (MSR145, MSR Electronics GmbH, Switzerland).

### Feed characterization

The weights of feed offered and feed refused were recorded on a daily basis. Representative sub-samples of the material offered each day were oven-dried at 80 °C to constant weight in order to determine dry matter (DM) content. A further sub-sample of the feed offered was collected at each feeding, bulked for each 3-day chamber period, and thoroughly mixed. Sub-samples were then taken to determine chemical and botanical composition. Sub-samples for chemical analysis were freeze-dried and milled through a 1 mm sieve. Ash was measured by igniting samples in a muffle furnace at 550 °C for 16 h. Gross energy (GE), included because these values were used in subsequent calculations of Y_m_ coefficients (CH_4_-E/GE intake), was determined by adiabatic bomb calorimetry (Gallenkamp autobomb; Sanyo Gallenkamp PLC, Loughborough UK). Total nitrogen concentrations were determined using a Leco FP 428 nitrogen analyser (Leco Corporation, St. Joseph, MI, USA), and were expressed as crude protein (nitrogen × 6.25). Water-soluble carbohydrate (WSC) concentrations were measured by an automated anthrone technique (Thomas 1977). Neutral detergent fibre (NDF) and acid detergent fibre were determined using the method of Van Soest *et al* (1991), adapted for the Gerhardt Fibrecap detergent system (FOSS UK Ltd, Warrington, UK). Digestibility of organic matter in the DM was determined using the two-stage pepsin-cellulase *in-vitro* method described by Jones & Hayward (1975). In order to characterize botanical composition a sub-sample from the bulked material from each experimental run was separated into the following categories: ryegrass, unsown grasses, white clover (*Trifolium repens*), other forbs and dead material. All separated material was then dried to constant weight at 80 °C and sward composition expressed on a proportional DM basis.

### Data analysis

Metabolic live weight (MLW) was calculated as mean LW^0·75^. Tier 1 (Dong *et al.* 2006) equivalent EFs (kg/year) were calculated as: CH_4_ (g/day) × 365. Two-way analysis of variance with a blocking structure of chamber run/(animal × period) was used to investigate forage type × breed type interaction effects (Genstat 16; VSN International Ltd, Hemel Hempstead, UK).

## RESULTS

### Composition of the forages offered

Proportionally the ryegrass sward was made up of 0·92 perennial ryegrass, 0·02 unsown grasses and 0·06 dead material. In comparison the permanent pasture was more diverse. Only 0·05 of this sward was accounted for by perennial ryegrass, with unsown grasses (mainly *Agrostis* spp., *Festuca* spp., *Poa* spp and *Holcus lanatus*) making up 0·77 of the sward. Clover accounted for just under 0·04 of the sward, and other forb species for 0·03. This sward also contained a higher proportion of dead material (0·12).

The DM content of the two forages offered was similar, as was the crude protein concentration (Table 1). However, compared with the low-input permanent pasture the ryegrass had a higher WSC concentration, lower fibre concentrations and a higher digestibility.

**Table 1.** Chemical composition of the forages offered. All values g/kg DM unless otherwise stated

	Ryegrass	Permanent pasture	s.e.d.	*P* value
DM (g/kg FM)	219	219	16·5	0·974
Ash	92	75	4·9	0·004
CP	133	118	10·7	0·190
WSC	226	118	17·1	<0·001
NDF	440	569	21·6	<0·001
ADF	242	318	11·3	<0·001
DOMD	731	593	24·7	<0·001
GE (MJ/kg DM)	17·3	17·7	0·14	0·040

DM, dry matter; FM, fresh matter; CP, crude protein; WSC, water-soluble carbohydrates; NDF, neutral detergent fibre; ADF, acid detergent fibre; DOMD, digestible organic matter in the DM; GE, gross energy.

### Role of breed type and pasture type on voluntary intake and methane emissions

Although the lambs of the two breed types had the same BCS, as expected the smaller, native WM lambs were significantly lighter than the TexX lambs at the start of the experiment despite being approximately the same age (Table 2). While total daily CH_4_ emissions were higher for the TexX lambs compared with the WM lambs, the yield of CH_4_ per unit of forage consumed was similar for the two breed types, as was the yield per unit MLW.

**Table 2.** Effect of breed type and pasture type on voluntary dry matter intake (DMI) and methane (CH_4_) emissions by weaned lambs (where WM, Welsh Mountain; TexX, Texel cross; MLW, metabolic live weight)

	Ryegrass		Permanent pasture			*P* value*	
	WM	TexX	WM	TexX	s.e.d.	Breed	Pasture
Weight of lambs at start (kg)	27	35	26	34	1·2	<0·001	0·307
DMI (g/day)	931	1041	705	758	74·7	0·275	<0·001
DMI (g/kg MLW)	78	73	61	53	4·7	0·168	<0·001
CH_4_ emitted (g/day)	15	17	12	14	1·1	0·035	<0·001
CH_4_ emitted (g/kg MLW)	1·2	1·2	1·0	1·0	0·07	0·651	<0·001
CH_4_ emitted (g/kg DMI)	16·1	16·7	16·7	18·8	0·87	0·116	0·004
CH_4_ emitted (kg/year)†	5·4	6·3	4·3	5·1	0·41	0·035	<0·001
CH_4_-E/GE intake (MJ/MJ)	0·052	0·054	0·053	0·059	0·0028	0·120	0·018

GE, gross energy

* There were no significant interaction effects.

† Estimated using the Tier 1 approach (Dong *et al.* 2006).

Pasture type had a highly significant effect on daily CH_4_ emissions (Table 2), in turn influencing the EFs estimated using the Tier 1 approach (Dong *et al.* 2006). Total output of CH_4_ per day was higher when offered ryegrass, but DMI was also higher on this forage type. The CH_4_ yield per unit of forage consumed and CH_4_ energy excreted per unit GE intake (Y_m_) were higher for lambs consuming the permanent pasture. No forage type × breed type interactions were identified.

## DISCUSSION

The current experiment explored the potential for breed type and pasture type to influence enteric CH_4_ emissions from growing lambs. Data were collected for weaned lambs offered material from swards representative of the pastures used by commercial producers at the corresponding stage of the sheep production cycle. There are relatively few previous studies that have reported emissions for sheep <1 year old, and the majority of these have been based on animals aged 7–12 months, i.e. store lambs rather than finishing lambs.

### Influence of breed type

While the potential to exploit within-breed variation in CH_4_ emissions within genetic improvement programmes has been recognized (Pinares-Patiño *et al.* 2011; Lambe *et al.* 2014), there is little information available regarding the extent to which CH_4_ emissions vary between different breeds of sheep. For the current experiment two contrasting breed types of lamb were chosen. The WM is a hardy hill breed adapted to survive on low-quality forage in the exposed conditions of upland Wales. They play an important role in the stratified sheep industry within the UK because as well as producing purebred hill lambs WM ewes are popular for crossing with longwool rams to produce the type of larger crossbred ewe (‘mules’ and ‘halfbreds’) commonly used for fat lamb production (Pollott 2014). The TexX lambs were representative of the output from this final cross, in which a terminal sire such as the Texel is used to produce prime lamb meat (Pollott 2014).

Overall, breed type was found to have a limited role in influencing CH_4_ emissions. While the TexX lambs produced more CH_4_ per day, the amounts produced by the two different breed types was similar on a per unit intake and per unit MLW basis. Recent experiments in which measurements were made on different breed types of hill (Aubry *et al.* 2014*a*) and lowland (Aubry *et al.* 2014*b*) replacement ewes aged from 8 to 19 months also found no difference in CH_4_ emissions as a proportion of feed intake. It was not possible to measure LW gain meaningfully within the current experimental design, but prime lambs such as the TexX lambs would be expected to be growing faster and reach slaughter weight earlier than purebred hill lambs (Carson *et al.* 2001). Consequently, although the CH_4_ emissions per day may be higher for these animals, emissions intensity in terms of CH_4_ per unit weight gain would be expected to be lower.

### Influence of pasture type

Studies with lambs to date have largely focussed on the effects of selected dietary ingredients and additives (Molano *et al.* 2008; Mao *et al.* 2010; Li *et al.* 2012, 2013; Avila-Stagno *et al.* 2013; Barnett & Hegarty 2014; El-Zaiat *et al.* 2014). The role of different pasture types has been limited to comparisons of grass and white clover (Hammond *et al.* 2011). The current study found that pasture type had a much stronger influence on CH_4_ emissions than lamb breed type. The pastures chosen for the experiment were typical of sward types grazed by comparatively intensively (re-seeded ryegrass) and extensively (permanent pasture) produced lambs during the post-weaning period. The main differences in nutritional value related to the concentrations of WSC and fibre within the forages, and related effects on digestibility. In turn these differences will likely have influenced voluntary feed intake, with the significantly lower DMIs for lambs offered the permanent pasture probably being due to longer rumen retention times (Thornton & Minson 1973). An increase in NDF concentration has been shown to increase CH_4_ yield (g CH_4_/kg DM intake) for dairy cattle (Yan *et al.* 2009), and the results obtained for growing lambs in the current trial reflect this relationship. In contrast, Hammond *et al*. (2011) found no difference in CH_4_ yield from older (1–2 years) sheep offered fresh white clover or perennial ryegrass, despite marked differences in chemical composition of the forages.

### Inventory and industry applications

The current approach to the UK GHG inventory is to assume the IPCC Tier 1 default EF for enteric fermentation for all mature sheep (i.e. >1 year old; Dong *et al*. 2006). Lambs have a lower average LW than mature sheep and the majority have a lifespan of <12 months, and would be expected to be associated with a lower EF than mature sheep. The UK therefore uses a country-specific EF for enteric fermentation for lambs at 40% of that of an adult sheep together with a reduction factor reflecting the reduced lifespan of lambs (Webb *et al.* 2013), which is estimated as 8·1 months. No adjustment is made for the pre-weaning period. The equivalent EFs calculated from the daily emission rates for the lambs in the current study are higher than the value estimated by Webb *et al*. (2013) (Table 3). In situations where finishing lambs are supplemented with concentrate feeds the CH_4_ emissions would be expected to be reduced, as such animals should produce less CH_4_ per day (Gill *et al.* 2010) and have a shorter time to finish. However, poor economic returns from sheep production mean that most farmers are keen to avoid the additional costs associated with supplementary feeding. The overall treatment mean for Y_m_ of 0·054 MJ/MJ is lower than the IPCC Tier 1 value for sheep of 0·065 MJ/MJ (Dong *et al*. 2006). This means that CH_4_ emissions for growing lambs are likely to be lower than currently calculated using the default IPCC Tier 1 methodology because a lower proportion of the GE consumed would be lost from the rumen as CH_4_.

**Table 3.** Estimated enteric fermentation emission factors (EF) for sheep. The ryegrass and permanent pasture values were generated based on measured mean emission rates per day for Welsh Mountain and Texel cross lambs aged 3–4 months old

	EF	Reference
*Published*		
Mature sheep (>1 year old)	8·0 kg CH_4_/head/year	Dong *et al.* (2006)
UK Lambs*	2·2 kg CH_4_/head/year	Webb *et al.* (2013)
*Current study*		
Ryegrass (for 12 month period)	5·9 kg CH_4_/head/year	
Permanent pasture (for 12 month period)	4·7 kg CH_4_/head/year	
Ryegrass (for 8·1 month lifespan)	4·0 kg CH_4_/head/year	
Permanent pasture (for 8·1 month lifespan)	3·2 kg CH_4_/head/year	

Methane, CH_4_

* Assumes emission factor is 0·4 of that of an adult sheep and the average lifespan of lambs is 8·1 months.

The current study provides an emissions benchmark against which mitigation targets can be set and progress measured. It also provides representative data for use in wider comparisons of environmental impact. For example, carbon footprinting is increasingly used in the food supply chain to estimate the quantity of GHG emitted at different stages of the production process (Edwards-Jones *et al.* 2009; Jones *et al.* 2014). Enteric CH_4_ emissions are estimated to contribute to over 40% of the mean footprint (Jones *et al.* 2014), but current calculations are based upon the default IPCC EFs. By refining input data and EFs the precision of carbon footprint models can be improved both spatially and temporally and uncertainty in the carbon footprint estimate reduced (Basset-Mens *et al.* 2009).

## CONCLUSIONS

Although total daily CH_4_ emissions were higher for the prime lambs compared with the smaller hill lambs when offered fresh forage, the yield of CH_4_ per unit intake was similar for the two breed types. The total output of CH_4_ per day was higher when offered ryegrass compared with permanent pasture, but CH_4_ emissions per unit intake and Y_m_ were higher on the permanent pasture. Overall the results indicate that forage type has a greater impact than breed type on CH_4_ emissions from weaned lambs.
